# The Evoked Potential Operant Conditioning System (EPOCS): a research tool and an emerging therapy for chronic neuromuscular disorders

**DOI:** 10.3791/63736

**Published:** 2022-08-25

**Authors:** N. Jeremy Hill, Disha Gupta, Amir Eftekhar, Jodi A. Brangaccio, James J. S. Norton, Michelle McLeod, Tim Fake, Jonathan R. Wolpaw, Aiko K. Thompson

**Affiliations:** 1. National Center for Adaptive Neurotechnologies, Stratton VA Medical Center, Albany, NY, USA.; 2. Electrical & Computer Engineering Department, State University of New York at Albany, NY, USA; 3. College of Health Professions, Medical University of South Carolina, Charleston, SC, USA; 4. Department of Biomedical Sciences, State University of New York at Albany, NY, USA

## Abstract

The Evoked Potential Operant Conditioning System (EPOCS) is a software tool that implements protocols for operantly conditioning stimulus-triggered muscle responses in people with neuromuscular disorders, which in turn can improve sensorimotor function when applied appropriately. EPOCS monitors the state of specific target muscles (e.g. from surface EMG while standing, or by gait cycle measurements while walking on a treadmill), and automatically triggers calibrated stimulation when pre-defined conditions are met. It provides two forms of feedback that enable a person to learn to increase or decrease the targeted pathway’s excitability. First, it continuously monitors ongoing EMG activity in the target muscle, guiding the person to produce a consistent level of activity suitable for conditioning. Second, it provides immediate feedback of the response size following each stimulation, and indicates whether it reached a target value.

To illustrate EPOCS use, this article describes a protocol through which a person can learn to decrease the size of the Hoffmann reflex—the electrically-elicited analog of the spinal stretch reflex—in the soleus muscle. Down-conditioning this pathway’s excitability can improve walking in people with spastic gait due to incomplete spinal-cord injury. The article demonstrates how to set up the equipment; how to place stimulating and recording electrodes; and how to use the free EPOCS software to optimize electrode placement, measure the recruitment curve of direct motor and reflex responses, measure the response without operantly conditioning, condition the reflex, and analyze the resulting data. It illustrates how the reflex changes over multiple sessions, and how walking improves. It also discusses how the system can be applied to other kinds of evoked responses to other kinds of stimulation (e.g., motor evoked potentials to transcranial magnetic stimulation), how it can address various clinical problems, and how it can support research studies of sensorimotor function in health and disease.

## SUMMARY

The Evoked Potential Operant Conditioning System (EPOCS) aids scientific investigation of sensorimotor function, and can administer targeted neurobehavioral training that can impact sensorimotor rehabilitation in neuromuscular disorders. This article describes its capabilities, and illustrates its application in modifying a simple spinal reflex to achieve lasting improvement in motor function.

## INTRODUCTION:

Over the past decade, *targeted neuroplasticity* strategies have emerged as a new approach to rehabilitation of neurological impairments.^1, 2^ One such strategy is operant conditioning of an evoked potential. This entails repeatedly eliciting electrophysiological responses that can be measured non-invasively—for example by electroencephalography (EEG) or surface electromyography (EMG)—and giving the person immediate feedback on the size of each response relative to a criterion level set by the therapist or investigator. Over time, this protocol trains the person to increase or decrease their response, and can consequently target beneficial change to a central-nervous-system site that is important in a behavior such as locomotion or reach-and-grasp. The targeted change benefits performance and, in addition, enables better practice that leads to widespread beneficial change that improves the entire behavior. For example, in people with incomplete spinal-cord injury (iSCI) in whom clonus impairs locomotion, operant conditioning that reduces the Hoffmann reflex in the soleus muscle of one leg improves locomotor muscle activity in both legs, and thereby lastingly increases walking speed and restores right/left step symmetry.^1, 3–5^ Another example is that paired-pulse stimulation can lastingly increase the size of the motor-evoked potential (MEP) to transcranial magnetic stimulation, thereby improving reach-and-grasp function in people with chronic hand and arm impairment following iSCI.^6^

Implementing such protocols demands special-purpose software that must perform multiple functions. Specifically, it must continuously acquire, process & save electrophysiological signals; it must continuously monitor the state of the nervous system and trigger stimulation appropriately under tight real-time constraints; it must provide continuous feedback, trial-by-trial feedback and session-by-session feedback; it must provide a user interface to guide setup and tuning by the investigator or therapist; finally, it must store and organize signal data and meta-information in a standardized format.

The Evoked Potential Operant Conditioning System (EPOCS) is our answer to this outstanding need. Under the hood, the EPOCS software is based on our open-source neurotechnology platform BCI2000,^7, 8^ which is used in hundreds of laboratories around the world. In EPOCS, the standard BCI2000 user interface is hidden, and replaced by a streamlined interface that is optimized for evoked-potential operant-conditioning protocols.

The current article and its accompanying video illustrate the use of EPOCS in one particular protocol: operant conditioning to reduce the size of the Hoffmann (or “H-”) reflex in the soleus muscle. This response is the electrically-elicited analog of the “knee-jerk” stretch reflex. H-reflex down-conditioning has been shown to reduce the impact of clonus on, and to thereby improve, locomotion in animals with iSCI^9–13^ and humans with iSCI, multiple sclerosis or stroke.^14, 15, 5^ It can be applied without adverse side-effects in animals and people with or without neurological injury.^16, 17^

**Figure 1:**
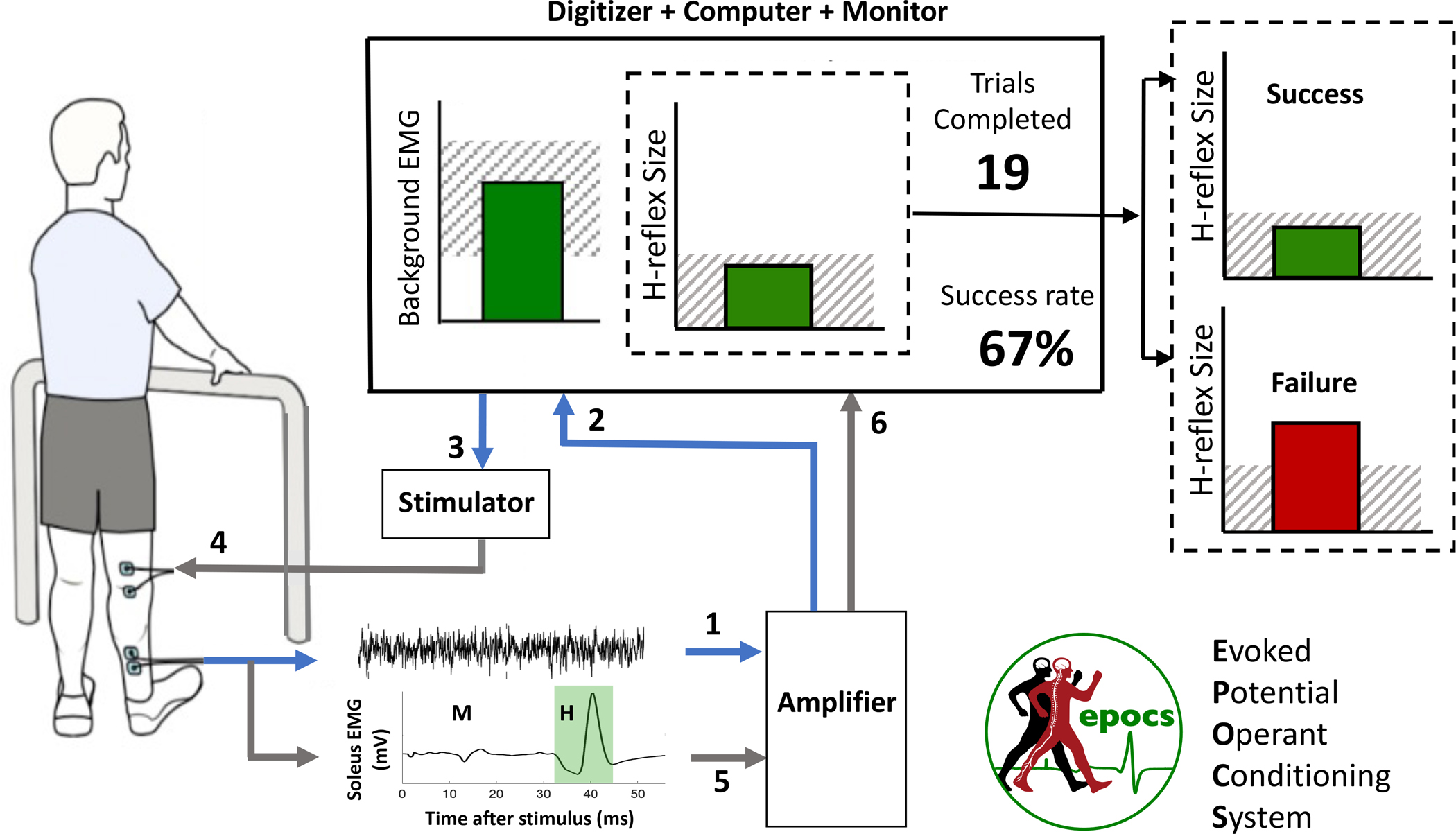
Schematic illustration of EPOCS’ core functionality during down-conditioning of the soleus H-reflex. The participant views a large monitor screen that shows the background EMG level, the most recent H-reflex size, the number of trials completed so far in the current run of 75, and the running proportion of successful trials for the run. The sequence of events in one trial is denoted by the numbers 1–6 as described in the Introduction.

The operant conditioning protocol functions by performing multiple *trials*, each lasting several seconds. The sequence of events of one trial is shown schematically in Figure 1, with numbers denoting the following functions:

1. Background (i.e., ongoing) EMG is recorded from bipolar surface electrodes over the target muscle (soleus) and its antagonist (tibialis anterior). Background level is evaluated as the mean rectified value of the high-pass-filtered signal in a sliding window.
2. Background EMG level in the target muscle is shown as the height of a bar, continuously updated on the participant’s screen. This helps the participant to keep the activity within a specified range (hatched region).
3. The software judges the appropriate moment for electrical stimulation, and triggers the stimulator accordingly. The principal criteria are: at least 5 seconds must have elapsed since the previous stimulation; and background EMG level must have remained in the specified range continuously for 2 seconds.
4. A constant-current stimulator delivers an electrical pulse transcutaneously to the tibial nerve (typically monophasic, with 1 millisecond duration).
5. The resulting stimulus-locked response is recorded. The software computes the sizes of two components of particular interest: the earlier M-wave which reflects muscle activation resulting from direct stimulation of the motor axon, and the later H-reflex which reflects the signal relayed through a reflex arc in the spinal cord.^18–22^ EPOCS refers to these as the *reference response* and *target response*, respectively.
6. H-reflex size for the current trial is displayed as the height of a second bar, relative to a desired criterion level that defines a successful or unsuccessful trial. For down-conditioning, the bar is dark green if the H-reflex size fell below the criterion, or bright red if it did not (vice versa for up-conditioning). Simultaneously, the numeric display of the cumulative success rate is updated accordingly. Together, these graphical display elements provide the immediate positive or negative reinforcement on which operant conditioning relies.^23^

A human H-reflex conditioning protocol typically consists of 6 baseline sessions, followed by 24–30 conditioning sessions spread over 10 weeks (i.e. 3 per week), and several follow-up sessions over the subsequent 3–6 months.^16, 14^ Each session lasts 60–90 minutes.

To support this protocol as well as other related protocols, EPOCS provides five distinct modes of operation, each served by one of the tabs of its main window, entitled Stimulus Test, Voluntary Contraction, Recruitment Curve, Control Trials and Training Trials.

In Stimulus Test mode, the software triggers a stimulus every few seconds, not necessarily contingent on the state of the target muscle. The response signals are shown on screen after each stimulus. This allows the operator to verify the quality of the electrode connections and the EMG signal; to optimize the position of the stimulating and recording electrodes; and to establish the individual’s response morphology.

In Voluntary Contraction mode, the software measures and shows background EMG level while the participant is encouraged to contract the muscle as much as possible, in the absence of electrical stimulation. In some protocols, the EMG level at maximum voluntary contraction (MVC) is a useful reference for setting background EMG criteria. In the particular protocol demonstrated here, this will not be necessary, as a stable standing posture standardizes the activity of the soleus muscle sufficiently.

In Recruitment Curve mode, stimulation is contingent on background EMG level (shown continuously on screen) remaining in the correct range; response signals are shown on screen after each stimulus; and the sequence of responses may be analyzed at the end of a run. This allows the operator to determine the start and end of the time intervals in which the responses of interest appear; to determine the relationship between stimulation intensity and response size, both before and after conditioning runs; and to determine the stimulation intensity to be used for conditioning.

In Control Trials mode, stimulation is contingent on background EMG level (shown continuously on screen), but no feedback is given about the target response size. The sequence and distribution of response sizes may be analyzed. This mode may be used to gather baseline measurements of response size, or as a control condition for comparison against operant conditioning in a crossover or between-subject experimental design. It can serve as a basis for setting the performance criterion for operant conditioning at the beginning of each session.

Finally, in Training Trials mode, stimulation is contingent on background EMG level (shown continuously on screen), and trial-by-trial reinforcement is also provided by showing the target response size, as described above and shown in Figure 1. This is the mode in which operant conditioning is performed.

The next section will guide the reader through the five modes by demonstrating the protocol for down-conditioning the soleus H-reflex in an adult participant without neurological injury.

## PROTOCOL:

All procedures described here were approved by the institutional review boards of the authors’ respective institutions. Note: terms in boldface indicate labels that should be visible on the hardware and/or in the software graphical user interface.

1. Install and configure the software. This step only has to be performed once for a given hardware configuration.

1.1. Go to https://neurotechcenter.org/epocs for instructions on obtaining the latest software installer.

1.2. Install the EPOCS software using the downloaded installer.

1.3. Ensure that the necessary drivers and software are installed for the digitizer. EPOCS requires a 64-bit installation of NI-DAQmx.

1.4. Launch the **NI-MAX** application, select the device to be used under **Devices and Interfaces**, and ensure that its **Name** is “Dev1”. Then under **Configure → Power-up States**, ensure that the **Line State** checkbox for port 0 line 7 is unchecked (zeroed). Also zero the corresponding **Tristate** checkbox, if there is one.

1.5. Using Windows’ **Add or Remove Programs** tool, remove any unnecessary software that might intermittently consume processor resources in the background, as this can lead to glitches in real-time signal processing. Make sure to remove any software update/troubleshooter suites provided by the computer’s manufacturer, as these have been known to cause serious performance problems (for system updates, just rely on Windows Update).

2. Set up the hardware.

2.1. Set up the digitizer, to coordinate input and output.

2.1.1. Using a short piece of solid-core insulated wire, patch the spring terminal for digital output port 0 line 7 (marked **P0.7**—or possibly **DIO7** on older equipment) to the spring terminal for **USER** output.

2.1.2. Attach a female/male/female BNC tee connector to the **USER** output.

2.1.3. Connect the **USER** output to the external trigger input port of the stimulator.

2.1.4. Connect the first and second amplified EMG signal cables to the first and second analog input channels of the data acquisition card, respectively. (These are marked **AI0** and **AI1**—or possibly **ACH0** and **ACH1** on older equipment.)

2.1.5. Make an additional connection from the **USER** output, back into the third analog input channel (marked **AI2** or **ACH2**).

2.2. Set up the constant-current stimulator. NOTE: To make the protocol generalizable to a variety of stimulator brands and models, this article describes manual stimulus intensity control rather than taking advantage of EPOCS’ support for automatic control of certain stimulator models.

2.2.1. Turn on the stimulator and configure it to deliver 1-millisecond monophasic pulses.

2.2.2. For the DS8R model: ensure that stimulus intensity is controlled by the front panel or USB interface, not via the rear analog input.

2.2.3. Plug in the long output cable and connect it to the snap leads that will attach to the stimulating electrode pads.

2.3. Set up the analog amplifier to deliver at least two EMG channels.

2.3.1. Turn on the amplifier.

2.3.2. Ensure all channel **GAIN** values are at their default value of 500, and the corresponding **VARIABLE** knobs are turned to their minimum value of 1.

2.3.3. Connect the portable unit to the amplifier using the long cable.

2.3.4. Insert two square 9-Volt batteries into the portable unit’s battery pack.

2.3.5. Strap the portable unit and battery pack around the participant’s waist.

3. Attach stimulation and recording electrodes in their starting positions.

3.1. Use any previously-noted landmarks or measurements to recreate previous participant-specific electrode positions as closely as possible.

3.2. Prepare the skin where electrodes will be attached by wiping with alcohol pads, to remove excess oil, then wipe with a paper towel to remove dead skin.

3.3. Attach stimulation electrodes in position to stimulate the tibial nerve precisely, with minimal effect on the common peroneal nerve.

3.3.1. Use the larger (22 × 35 mm) electrode as the anode; place it at the apex of the popliteal fossa where the sciatic nerve branches into tibial and common peroneal nerves.

3.3.2. Place the cathode (22 × 22 mm) at the crease of the knee, directly below the anode with a 3–4 cm separation between electrode centers.

3.4. Attach EMG recording electrodes in a bipolar montage at the target muscle (soleus) as follows.

3.4.1. To determine the correct location, first find the gastrocnemius muscle by palpating while the participant alternates betweening standing on their toes and standing naturally.

3.4.2. Place the first electrode directly below the distal border of the gastrocnemius muscle belly.

3.4.3. Place the second electrode below the first, with a 5 cm spacing between electrode centers. Keep both electrodes in line with the Achilles tendon.

3.5. Attach EMG recording electrodes in a bipolar montage at the antagonist muscle (tibialis anterior).

3.5.1. Identify the muscle by having the participant lift (dorsiflex) their toes.

3.5.2. Place electrodes on the muscle belly, about 1/3 of the way down from the fibular head to the ankle, with a 5-cm vertical separation between electrode centers.

3.6. Attach a ground electrode at the patella.

3.7. Connect the EMG amplifier leads:

3.7.1. Plug the green-taped active electrode into channel **1** on the portable unit, and connect the red clips to the target-muscle electrodes (soleus) and the green clip to the ground electrode.

3.7.2. Plug the black-taped active electrode into channel **2** on the portable unit, and connect the clips to the antagonist-muscle electrodes (tibialis anterior).

3.8. Connect the battery pack to the portable unit.

3.9. Connect the stimulation snap leads to the stimulation electrodes.

4. Use the EPOCS software.

4.1. Position the monitor such that both investigator and subject (or therapist and patient) can see it clearly.

4.2. Launch an EPOCS session. (NOTE: A session is defined as one visit to the laboratory or clinic, typically lasting 60–90 minutes.)

4.2.1. Double-click the **EPOCS** icon to launch the application.

4.2.2. Enter the participant ID code (or choose from the list of previously-used IDs).

4.2.3. If this is a continuation of an existing session (for example, if the software had to be re-launched following an interruption), press **Continue Session**. This will only be available if a session for the specified participant has been started in the last three hours.

4.2.4. Otherwise press **Start New Session**. This will create a new data folder, date- and time-stamped and marked with the participant ID.

4.3. Verify electrode location and contact quality, and adjust as necessary.

4.3.1. Ensure that the **Stimulus Test** tab is displayed.

4.3.2. In **Settings** → **Stimulation**, configure the **Min. interval for Stimulus Test** to 3 sec. Note that this is configured separately from the **Min. interval for normal usage**, which will typically be longer (5 sec).

4.3.3. Leave the **Digitimer Link** setting disabled. NOTE: When enabled, this would allow software control of stimulation intensity when using certain stimulator models. The current protocol will instead demonstrate *manual* control of stimulus intensity, which is applicable across multiple stimulator makes and models.

4.3.4. On the stimulator control panel, set the stimulus intensity to 5 mA and enable stimulation.

4.3.5. Ask the participant to stand, with their hands partially supporting their weight on a walker if necessary.

4.3.6. Warn the participant to expect stimulation, then press **Start** to start a new run (i.e., to start recording signals continuously to a new file). Runs will be numbered sequentially and their files will never overwrite each other.

4.3.7. Each repetition of stimulation is called a trial. The EMG responses elicited in each trial are shown immediately. Assess the M-wave and H-reflex elicited in the target muscle (the upper, blue trace) and antagonist muscle (the lower, red trace).

4.3.8. If necessary, increase the current gradually (to 10 mA or more, as necessary) until the responses appear clearly.

4.3.9. To find the optimal location for stimulating (i.e. the location that yields the largest H-reflex), move the cathode a full-electrode width medially and then laterally, followed by half-electrode width medially and then laterally, and finally by a full-electrode width up and then down.

4.3.10. Mark, note and photograph the position of the electrodes, to aid re-positioning in future sessions. It can even be helpful to make a plaster cast of the calf and back of the knee, and make holes in the cast that allow marks to be re-applied precisely.

4.3.11. Once the optimal positions for the electrodes have been found, replace repositioned electrodes with fresh electrodes.

4.4. For soleus conditioning, skip the **Voluntary Contraction** tab. (The **Background EMG** range setting will instead be set around the person’s natural weight-bearing standing level.)

4.5. Measure the maximum M-wave and H-reflex sizes (M_max_ and H_max_) by charting the relationship between stimulus intensity and response. This relationship is described by the *recruitment curve*, which should be measured before and after control or training trials in each session. In the first session, the recruitment curve will guide selection of an appropriate stimulation intensity for use throughout the conditioning process.

4.5.1. Switch to the **Recruitment Curve** tab.

4.5.2. In **Settings** → **EMG**, configure the ranges that the target and antagonist background EMG avs must stay within to enable stimulation. The upper or lower limits may be left blank if no corresponding restriction is to be imposed. The **Background Hold Duration** setting dictates how long the participant must continuously keep the EMG in these ranges, to trigger each stimulus.

4.5.3. Enable the stimulator, and set the intensity to 5 mA (the minimum value to be used in recruitment curve measurement). (NOTE: this value is an example, and should be chosen on a case-by-case basis—see Discussion).

4.5.4. In the participant’s first session, allow them to practice keeping the EMG in the correct range for the required duration:

4.5.4.1. With the participant standing, press **Start**.

4.5.4.2. Demonstrate to the participant how the background EMG level in the target muscle is shown in real time as the height of the bar, against a shaded region that shows the target range.

4.5.4.3. Explain to the participant that activity from both muscles (target and antagonist) must be within their required ranges to turn the bar from a bright red to a darker green (although the antagonist activity level is not shown directly).

4.5.4.4. To adjust the background ranges: press **Stop** followed by **Settings**; then enter the new numbers, press **OK**, then **Start** again.

4.5.4.5. Press **Stop** when the practice run is finished.

4.5.5. To measure the recruitment curve: with the participant standing, press **Start**.

4.5.6. If the H-reflex is already visible at the chosen starting intensity, gradually decrease the current until the H-reflex is no longer seen. Then **Stop** and re-**Start** the run.

4.5.7. Pay close attention to the **Trials Completed** counter. After every four trials, manually increase stimulation intensity by 2 mA. (NOTE: this value is an example, and should be chosen on a case-by-case basis—see Discussion).

4.5.8. Continue until M-wave size reaches a plateau, provided the participant does not report discomfort.

4.5.9. Press **Stop** when done, and invite the participant to sit down to rest.

4.5.10. Make a note of the stimulus intensity values used for each trial. Associate any written records with the run number shown in the top right of the window. NOTE: If stimulation intensity is being controlled manually, this information will *not* be recorded by the software. At the end of any run, this information may be manually entered into the session log, along with any other notes, via the **Log** tab.

4.5.11. Press the **Analysis** button to open the analysis window and allow definition of the M and H waves.

4.5.11.1. In the upper pane of the analysis window, examine the stimulus-locked overlay of the target-muscle signals from each trial in the last run.

4.5.11.2. Use the mouse to adjust the beginning and end of the brown “reference” and green “target” intervals (in the H-reflex operant conditioning protocol, these correspond to M-wave and H-reflex, respectively).

4.5.11.3. When the intervals are correct, press the red **Use Marked Timings** buttons to save these personalized interval settings for future analyses.

4.5.12. In the **Sequence** pane in the lower half of the analysis window, assess the resulting recruitment curve. Adjust the settings to view either peak-to-peak or mean-rectified amplitude, and to pool results from consecutive trials (since the stimulus current was increased every four trials, specify **Trials to Pool:** 4). Record the resulting M_max_ and H_max_.

4.5.13. If this is the participant’s first session, optimize the target-muscle EMG recording locations:

4.5.13.1. Move the soleus electrodes medially by a half-electrode-width (or full electrode width if there is space on the leg). Then repeat the steps above to gather a full recruitment curve, and record the resulting M_max_ and H_max_.

4.5.13.2. Move the soleus electrodes the same distance laterally from their original position, and again perform a recruitment curve measurement to estimate M_max_ and H_max_.

4.5.13.3. Adopt the electrode positioning that maximizes H_max_: mark, note and photograph their positions as in step 4.3.10.

4.5.14. Choose a stimulus intensity that elicits a close-to-maximal H-reflex—ideally on the ascending (left-hand) slope of the H-reflex recruitment curve—but with the constraint that there must be a visible M-wave. Set this stimulus intensity value on the stimulator, and note it for future sessions. Also note the corresponding M-wave size (see Discussion).

4.6. Measure the distribution of H-reflex sizes without giving response feedback.

4.6.1. Switch to the **Control Trials** tab.

4.6.2. With the participant standing, press **Start**. As before, the participant’s goal is to use the feedback provided by the rising and falling bar to maintain a level of background muscle activity within the required range.

4.6.3. If this is a baseline or follow-up session, perform 75 trials in succession at the chosen stimulus intensity; if this is a conditioning session, perform just 20 trials. After the prescribed number of trials, press **Stop** to end the run.

4.6.4. Press **Analysis**. As before, an overlay of trial-by-trial response waveforms is shown in the upper panel, and the **Sequence** of response sizes below. This time, however, a new tab called **Distribution** is activated by default, on top of **Sequence.** It shows the distribution of H-reflex sizes, with summary statistics on the right.

4.6.5. Press **Log Results** to append the summary statistics to the session log.

4.6.6. If this is a baseline session, repeat the above steps for a total of 3 runs of 75 trials each. Then skip to the closing recruitment curve measurement (step 4.8).

4.6.7. If this is a conditioning session: to set an up-conditioning or down-conditioning criterion for subsequent operant conditioning runs:

4.6.7.1. Set the Target Percentile to 66. NOTE: In a down-conditioning protocol, this means that a successful trial is defined as one in which the response size is in the bottom 66% of the previously measured distribution; conversely, in up-conditioning, success means producing a response size in the top 66% of the distribution. These two criterion levels, along with the median, are shown by the vertical red lines. Either criterion may be adopted for conditioning, by pressing the **Up-Condition** or **Down-Condition** button.

4.6.7.2. Press **Down-Condition** to adopt the down-conditioning criterion. This action will be logged automatically and the analysis window will close.

4.7. Perform operant conditioning

4.7.1. Switch to the **Training Trials** tab.

4.7.2. With the participant standing, press **Start**.

4.7.3. If the participant has not seen it before, draw their attention to the new feedback bar in the middle of the screen. Explain that it shows the most recent H-reflex size relative to the hatched target range. If the response falls within the target range, the trial will be counted as successful and the bar will be dark green. If it falls outside the range, the trial will be counted as unsuccessful and the bar will be a brighter red.

4.7.4. Throughout the run, motivate the participant to perform as many successful trials as possible. The number of trials performed, and the proportion of trials that were successful in the run so far, are shown on the right of the screen.

4.7.5. After 75 trials, press **Stop** to end the run.

4.7.6. Press the **Analysis** button. The analysis window looks the same as it did for **Control Trials**. Again, use the **Sequence** tab to verify that the M-waves remained constant at the desired size.

4.7.7. As before: with the **Distribution** tab selected, use the **Down-Condition** button to update the operant conditioning criterion for the next run.

4.7.8. Repeat the operant conditioning procedure twice more, for a total of three runs of 75 trials each.

4.8. At the end of the session, perform another **Recruitment Curve** measurement (steps 4.5.1 through 4.5.9).

4.9. Finish the session

4.9.1. Type up any additional session notes in the **Log** tab. The log is saved automatically as it is filled in, in a date-stamped plain-text file in the session-specific data directory.

4.9.2. Close the EPOCS window. Data and logs will already have been saved.

4.9.3. To revisit the **Analysis** window for previously-recorded data, double-click the **EPOCS Offline Analysis** icon, and select the data file for the run to be analyzed. Wait for the raw signals to be processed (this may take a minute or more). NOTE: Data are saved as **.dat** files in BCI2000 format. The file name indicates the date and time of the session, the participant ID, the mode (ST for Stimulus Test, VC for Voluntary Contraction, RC for Recruitment Curve, CT for Control Trials and TT for Training Trials), and the sequential run number.

5. Repeat the session.

5.1. Schedule a total of 6 baseline sessions, 24 conditioning sessions (or 30, for people with neurological impairment) and 4 follow-up sessions. Schedule the baseline and conditioning sessions at a rate of 3 per week, each session lasting no longer than 90 minutes. Arrange for all sessions to be conducted at the same time of day, to minimize the effects of diurnal variation.

5.2. In each of the 6 baseline sessions, conduct an initial **Recruitment Curve** run, 3 runs × 75 **Control Trials**, and a final **Recruitment Curve** run.

5.3. In each of the 24 (or 30) conditioning sessions, conduct an initial Recruitment Curve, 1 × 20 **Control Trials**, 3 × 75 **Training Trials**, and a final **Recruitment Curve.**

5.4. Conduct 4 follow-up sessions at 10–14 days, 1 month, 2 months and 3 months after the last conditioning session. Depending on the goals of the study, these may be identical to baseline sessions, or to conditioning sessions.

## REPRESENTATIVE RESULTS:

**Figure 2:**
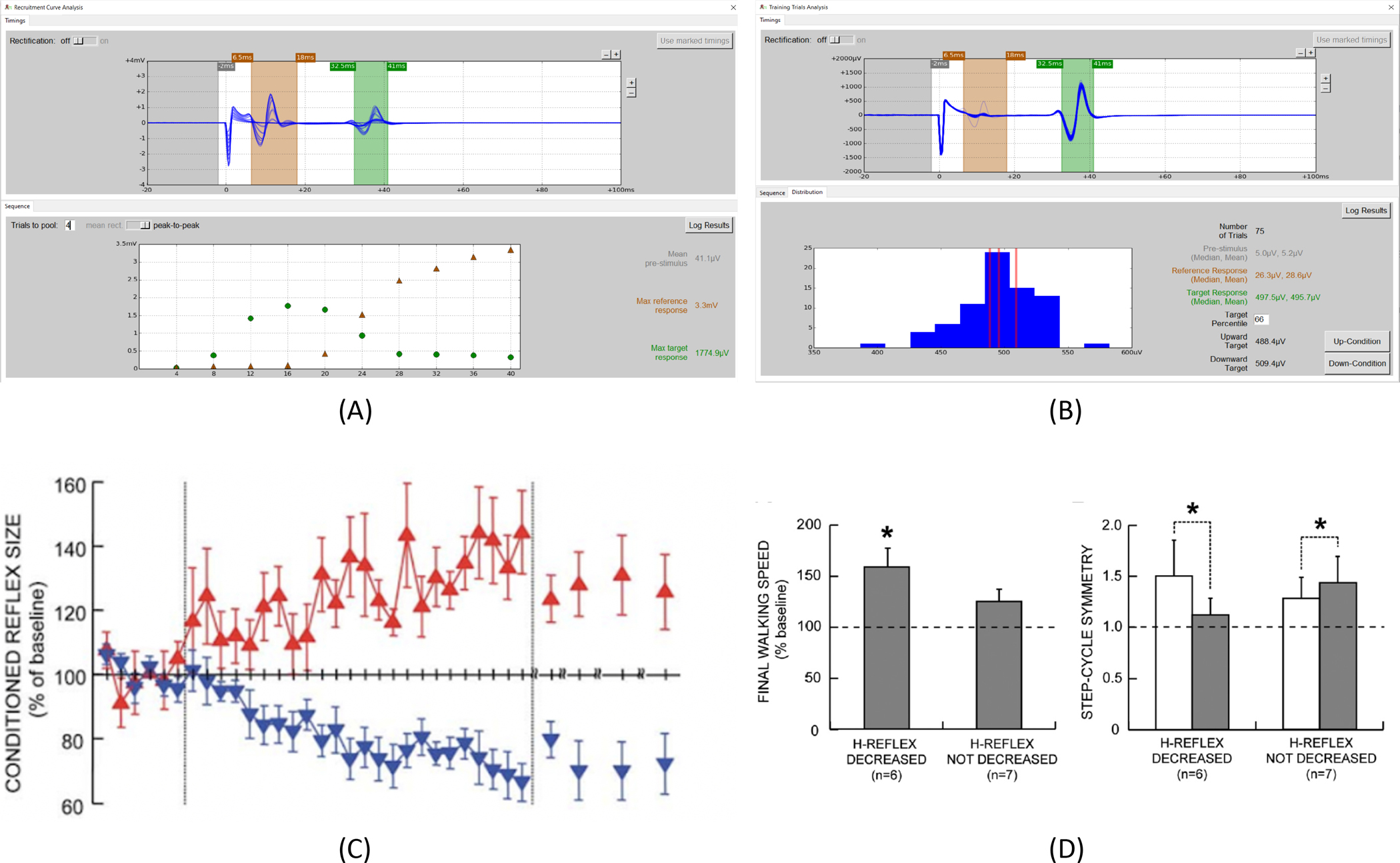
Representative results. (A): Screenshot of the **Recruitment Curve** analysis window in EPOCS. (B): Screenshot of the **Control Trials** or **Training Trials** analysis window in EPOCS. (C): Contrasting effects of up- and down-conditioning of the soleus H-reflex in uninjured participants (reprinted with permission from the Journal of Neuroscience^16^). (D): Therapeutic effect of soleus H-reflex down-conditioning on walking speed and gait symmetry in people with chronic impairment following incomplete spinal-cord injury (reprinted with permission from the Journal of Neuroscience^14^).

In Figure 2, panel (A) shows a screenshot of the EPOCS **Analysis** window following a run performed in **Recruitment Curve** mode during H-reflex operant conditioning (see protocol step 4.5). In the lower half of the window (**Sequence** pane), the horizontal axis shows trial number—hence, stimulus intensity increases from left to right. H-reflex size (green circles) rises, then falls as a function of stimulus intensity, whereas M-wave size (brown triangles) rises, then saturates. Panel (B) shows a screenshot of the EPOCS **Analysis** window following a run performed in **Control Trials** or **Training Trials** mode during H-reflex operant conditioning (see protocol steps 4.6 and 4.7). In the lower panel (**Distribution** pane), the histogram of H-reflex sizes facilitates selection of an appropriate criterion level for subsequent up- or down-conditioning. In panel (C), H-reflex size in uninjured participants is plotted as a function of session number across 6 baseline sessions, 24 conditioning sessions, and 4 follow-up sessions. Red upward triangles show mean H-reflex size (±SE) from N=6 successfully up-conditioned participants (out of 8); blue downward triangles show mean responses (±SE) from N=8 successfully down-conditioned participants (out of 9)—see Thompson et al. (2009) for further details.^16^ Panel (D) shows the beneficial effect of soleus H-reflex down-conditioning in participants with chronic lower limb impairment following incomplete spinal cord injury. The bars contrast results for N=6 participants whose H-reflex was successfully down-conditioned, against N=4 participants from the control condition (no operant conditioning) and N=3 participants in whom the down-conditioning protocol failed to reduce reflex size. Successful conditioning was associated with an improvement in gait symmetry, and in walking speed relative to baseline—see Thompson et al. (2013) for further details.^14^

## DISCUSSION:

The protocol described above is suitable for demonstrating soleus H-reflex down-conditioning in a typical adult without neurological impairment. The precise parameter values may vary from person to person, and particularly as a function of impairment. Whereas the participant’s recruitment curve reached M_max_ at a stimulating current of around 25 mA in the video, another person might require 50 mA or more, so the current would be increased in larger steps during recruitment curve measurement. A longer pulse duration may also be required. A third person might be more sensitive, and require smaller current settings. The protocol will also need to be adapted according to the muscle that is being conditioned. For example, when targeting the flexor carpi radialis muscle^24, 25^: a lower current setting is generally used; the **Voluntary Contraction** mode should be used to establish a scale for the background-EMG limits; and greater care must be taken both during optimization of electrode placement, and during optimization of posture, which must then be kept constant across trials.

The protocol is sensitive to variations in the relationship between stimulator current setting and the amount of current actually delivered to the nerve—this may be affected by small variations in posture, hydration of the participant, and drying out of the adhesive electrode gel. In H-reflex conditioning, this problem can be mitigated by using M-wave size as an indicator of effective stimulation intensity. It reflects the number of soleus motoneuron efferent axons excited by the stimulus. Thus, if M-wave size is kept constant, it implies that the number of primary afferent axons excited by the stimulus (i.e., the axons that elicit the H-reflex) is also kept constant (see also Crone et al., 1999^26^). Hence, this M-wave is referred to as the “reference” response in the software. For this reason, step 4.5.14 mentions that the target M-wave size should be recorded: it is actually more important to keep this response size roughly constant than to keep the nominal current strictly constant. The **Sequence** tab of the EPOCS **Analysis** window allows retrospective verification of M-wave constancy over each run; for soleus H-reflex conditioning, this is often sufficient to correct any problems. For greater control, a second monitor may be attached to the computer, where EPOCS can provide real-time M-wave analytics to inform trial-by-trial manual adjustment. Automation of this control task is an ongoing project.^27^

Diurnal variation may also affect a person’s electrophysiological responses.^28–31^ For this reason, it is recommended that all sessions be performed at the same time of day (i.e., within the same three-hour time window).

The success of operant conditioning may be sensitive to the accuracy of the time interval chosen by the operator to define the H-reflex; in particular, the interval should not be too wide. Detailed guidelines for correct interval definition are beyond the scope of the current article. This is also a function that will be automated in future versions of the software.

A critical step in the protocol is 4.5.7, in which the operator manually increases the stimulator current repeatedly after each fixed number of trials. Mis-counting the trials here, or mis-adjusting the current dial, can lead to distortion of the resulting recruitment curve. This possibility of user error can be mitigated by enabling the **Digitimer Link** option, which allows automation of the current adjustment for one particular stimulator model.

This article has focused on H-reflex conditioning, as it is the most fully developed of the potential clinical applications of EPOCS. The existing software helps researchers in the ongoing efforts to hone this particular protocol toward wide clinical dissemination.^32^ Beyond H-reflex conditioning, EPOCS may also be applied in its current form to a wider variety of stimulation methods and evoked responses. For example, it can equally well trigger a mechanical device that elicits a stretch reflex, which may also be conditioned.^33–35^ The approach is adaptable to an individual’s impairments: in one person, down-conditioning the soleus H-reflex improves locomotion by reducing spastic hyperreflexia^14^; in another, up-conditioning the tibialis anterior MEP improves locomotion by alleviating foot drop.^36^

While efforts are ongoing to produce a commercial implementation of the protocol, the original EPOCS software will be maintained in parallel as a research tool, to provide the necessary flexibility to expand the field of targeted neuroplasticity. This flexibility is enabled by the modularity and extensibility of the widespread and well-established BCI2000 software platform, on which EPOCS is based. This means that, with minimal intervention by a software engineer familiar with BCI2000, the system is re-configurable for an even wider variety of research purposes. For example, it can be configured to record additional biosignal channels or additional sensors for later analysis (e.g. foot switches and motion tracking sensors, for conditioning during locomotion). It can also be programmed to consider additional triggering criteria for stimulation (e.g., triggering stimulation only at a particular part of the gait cycle); or to trigger additional reinforcement stimuli on successful or unsuccessful trials. Example customization files are provided, using BCI2000’s scripting syntax.

Targeted neuroplasticity is still in its infancy. Its as-yet unexplored avenues are expected to provide great benefits both for developing novel therapeutic approaches (as discussed above), and for elucidating the natural history of disease, and mechanisms of central nervous system function in both health and disease.^2, 32, 37^ We are therefore committed to maintaining and supporting EPOCS as a key tool for realizing this therapeutic and scientific potential.

## Figures and Tables

**Table:** List of equipment and materials for an example EPOCS setup

Name of Material/Equipment	Company	Catalog Number	Comments/Description
Alcohol swabs	any		For application to skin
BNC cable (long) × 1	any		Male BNC to male BNC, long enough to reach from digitizer to stimulator
BNC cable (medium) × 2	any		Male BNC to male BNC, long enough to reach from amplifier to digitizer
BNC cable (short) × 1	any		Male BNC to male BNC, short (to patch between two digitizer ports)
BNC tee connector	any		Female-male-female BNC splitter
Computer	Lenovo	ThinkStation P340	A wide range of computing hardware is suitable, especially if using a USB digitizer (no PCI slots needed). Must run Windows 7 or later. Include standard keyboard & mouse.
Constant-current stimulator	Digitimer Ltd.	DS8R	The DS8R enjoys EPOCS automation support. If controlled manually, other constant-current stimulators may be used provided they have an external TTL trigger and can achieve a pulse duration of 1 ms or more.
Digitizer (option A)	National Instruments	USB-6212	USB digitizer with integrated BNC connectors.
Digitizer (option B)	National Instruments	PCIe-6321	PCIe digitizer—requires desktop computer with a free PCI slot, also cable and BNC terminal block (below). Not required if you bought Option A instead.
Digitizer cable (for option B only)	National Instruments	SHC68-68-EPM	Connects PCIe digitizer to BNC terminal block
Digitizer terminal block (for option B only)	National Instruments	BNC-2090A	19-inch-rack-mountable BNC terminal block
EMG amplifier system (analog)	Bortec Biomedical Ltd.	AMT-8	Analog amplifier + portable unit + long transmission cable + battery pack + two 500–gain active electrode leads (1 bipolar, 1 bipolar with ground)
Monitor	any		Large enough for the participant to see clearly from the intended viewing distance.
NeuroPlus electrodes (22 × 22 mm) × 6	Vermont Medical Inc.	A10040-60	Disposable self-adhesive silver/silver-chloride 22 × 22 mm surface-EMG electrodes. 6 needed per session (11 on participant’s first session)
NeuroPlus electrode (22 × 35 mm) × 1	Vermont Medical Inc.	A10041-60	Disposable self-adhesive silver/silver-chloride 22 × 35 mm surface-EMG electrode. 1 needed per session.
Snap lead × 2	any	EDR1220	Leads for stimulating electrodes: 1.5mm DIN to button snap
Wire	any		8–10 cm length of single-core insulated wire
